# App-based oral health promotion interventions on modifiable risk factors associated with early childhood caries: A systematic review

**DOI:** 10.3389/froh.2023.1125070

**Published:** 2023-03-10

**Authors:** Kamalapriya Ajay, Liane B. Azevedo, Anna Haste, Alexander John Morris, Emma Giles, Banu Preethi Gopu, Murali Perumbakkam Subramanian, Fatemeh Vida Zohoori

**Affiliations:** ^1^ School of Health and Life Sciences, Teesside University, Middlesbrough, United Kingdom; ^2^ School of Human and Health Sciences, University of Huddersfield, Huddersfield, United Kingdom; ^3^ Centre for Applied Psychological Science, Department of Psychology, School of Social Sciences, Humanities & Law, Teesside University, Middlesbrough, United Kingdom; ^4^ School of Dentistry, Institute of Clinical Sciences, College of Medical and Dental Sciences, University of Birmingham, Birmingham, United Kingdom; ^5^ Population Health Sciences Institute, Faculty of Medical Sciences, Newcastle University, Newcastle, United Kingdom

**Keywords:** mHealth, pre-schoolers, oral health, smartphone application, childhood caries

## Abstract

**Background:**

Early childhood caries (ECC) is a preventable chronic disease. Parents’ knowledge and attitudes toward oral healthcare have been associated with higher caries experience in their children. Mobile apps within the context of mHealth interventions are a potential tool for raising awareness and informing parents about their children's oral health.

**Objectives:**

The aim of this systematic review was to examine the effectiveness of mobile health apps, targeted at parents and caregivers, for the prevention of ECC.

**Data sources:**

A systematic search was carried out in five scientific databases; Embase, CINAHL, MEDLINE, PsycINFO and Web of Science.

**Study selection and data extraction:**

Original studies, delivering oral health interventions to parents of children <6 years *via* smartphones, were included. Both quantitative and qualitative findings from the included studies were extracted.

**Synthesis:**

A convergent segregated approach was used to integrate the quantitative and qualitative evidence, followed by side-by-side display and narrative synthesis.

**Results:**

Out of 5,953 retrieved articles, five met the inclusion criteria and were included in the review. Three articles reported quantitative findings, while two reported both quantitative and qualitative findings. Four studies reported that a mobile app can be an effective tool to improve the oral health knowledge of parents/caregivers, aiding them in incorporating good oral health habits into their children's daily routines.

**Conclusion:**

This review demonstrated that oral health promotion programs delivered through mobile apps to parents could be effective in improving child oral health awareness among parents. There is a need for more high-quality studies with a large number of participants to find out which features of mHealth interventions with parents could effectively be employed to reduce the prevalence of ECC. Further studies and apps should be developed based on evidence-based behaviour change techniques and incorporate features such as gamification to increase the effectiveness and engagement of the target population.

**Systematic Review Registration:**

[https://www.crd.york.ac.uk/prospero/display_record.php?], identifier [CRD42021268331].

## 1. Introduction

Early childhood caries (ECC) is defined as the presence of one or more decayed (non-cavitated or cavitated lesions), missing or filled (due to caries) surfaces in any primary tooth of children under 6 years of age (1). Despite being preventable, ECC remains a severe global oral-health problem (2). The prevalence of ECC varies among different groups of individuals and exceeds almost 85% in certain groups of underprivileged children (3). It is estimated to affect 621 million children globally, posing a particular problem for public health systems because of the repercussions such as chronic pain, and infections and ultimately leading to tooth extraction (4). The risk factors for ECC are multi-factorial such as microbiological, psychosocial, socio-demographic, and behavioural (5). However, with nearly all oral-health risk factors being modifiable, ECC can be prevented with the right strategies.

Several public health programmes have been implemented to monitor and prevent ECC in children. It is recognised that most ECC arises before the child begins school (6). Delivering oral-health education to parents/guardians and caregivers and consequently establishing good eating habits and access to fluoridated water are some approaches to ECC prevention (7). Parents’ knowledge and attitudes towards oral healthcare have been associated with higher caries experiences in their children (8). Data from a Cochrane review shows that educating parents/guardians on oral health could be an efficient way to reduce the prevalence of dental caries (9).

The widespread use of information and communication technology (ICT) has allowed individuals to make informed decisions about their health. Various devices such as computers, tablets, mobile/smartphones and satellite communications are used to deliver ICT interventions and are collectively referred to as “e-health interventions” (10). Due to the availability and adaptability of mobile phones and other portable devices such as tablets, they are frequently used for health services and delivering health information. Evidence suggests that mobile health (mHealth) interventions have great potential to improve the self-management of non-communicable diseases such as diabetes, hypertension, and obesity (11, 12). A systematic review, in 2013, concluded that oral-health education is effective in changing oral-health attitudes when implemented using electronic mediums as part of dental health programs (13). Similarly, a systematic review of mHealth interventions in the promotion of oral hygiene advice for children and adolescents found mobile phones to be an effective tool in encouraging orthodontic patients to follow oral hygiene advice (14).

Mobile apps within the context of mHealth interventions are also a potential tool for educating parents about their children's oral health, as supported by several randomised control trials (15–17). Majority of the dental caries prevention apps focus on providing oral hygiene education and information for different age groups including children or adults (18–20). However, a recent review (18) identified a significant gap in the availability of apps that are specifically designed to support parents of young children in preventing dental caries. The latter review (18) also found no examples of apps targeting ECC despite these also being preventable. The lack of evidence for this specific audience highlights the need for further development and implementation of apps that cater to the unique needs of parents when it comes to dental caries prevention in young children.

Although there exists a considerable number of trials that have assessed the usability and success rate of mobile apps in improving oral health in children under the age of six (15–17, 21, 22), to the best of our knowledge the findings from these studies have not been synthesised systematically. Therefore, this systematic review aimed to examine the effectiveness of mobile health apps, targeting parents and caregivers, for the prevention of ECC. This systematic review was guided by two research questions: (1) What types of mobile health applications are available to parents and carers to minimise the modifiable risk factors associated with ECC?; and (2) How effective are mobile health applications for parents and carers in modifying risk factors for ECC?

## 2. Methods

The review was conducted in accordance with the Joanna Briggs Institute (JBI) manual for evidence synthesis (23) and has been reported using the Preferred Reporting Items for Systematic Reviews and Meta-Analyses (PRISMA) criteria (24). The protocol for this review was registered with the International Prospective Register of Systematic Reviews (PROSPERO) (registration no. CRD42021268331).

### 2.1. Study selection

#### 2.1.1. Inclusion criteria

Studies were included if they fulfilled the following inclusion criteria:

• **Study Design**: Quantitative and qualitative primary studies, Randomised Control Trials (RCTs), Controlled Clinical Trials (CCTs), and secondary data analysis studies observational, qualitative and mixed-methods studies.
• **Publication Period and Language**: From 1996 onwards and limited to articles published in English.
• **Population**: Parents and caregivers with children ≤6 years of age.
• **Intervention**: Interventions delivered using mobile phone applications to increase positive attitudes and promote oral-health behaviours to prevent ECC in children by targeting parents and/or caregivers.
• **Comparator/Control**: In studies with a comparative control group, comparators were the population who receive usual care/no intervention.
• **Outcomes**: Since this is a mixed-methods systematic review a range of quantitative and qualitative outcomes were included:
  ○ *Quantitative outcomes*: Studies that have reported clinical outcomes: caries experience, dental plaque, measure of treatment of ECC such as number of extractions under general anaesthesia, pain caused by dental caries measured using validated scales, and number of sepsis episodes caused by dental caries. Studies reporting behavioural change outcomes and proxy behavioural change outcomes.
  ○ *Qualitative Outcomes*: Studies reporting qualitative outcomes of experiences, attitudes and beliefs of parents and caregivers towards using mobile apps which target oral-health behaviour.

### 2.2. Search strategy

An initial scoping search using keywords was conducted in PubMed. Selected studies that were retrieved from the scoping search were used to develop a detailed search strategy (Supplementary File S1). The following five databases were searched using the final search strategy on June 2021: Embase using the OvidSP platform; CINAHL and MEDLINE using the EBSCO host platform, PsycINFO and Web of Science. References of previously published review articles were also checked for eligibility.

### 2.3. Data management and study selection process

All identified records were uploaded into EndNote X8.2 (25), with records from different databases being combined and duplicates removed. Studies were then uploaded to Rayyan.ai software (26) for the title and abstract screening. A pilot screening of titles and abstracts (10% of a sample) was performed by KP, BP, and MK to maximise efficiency and minimise review errors before embarking on full screening. The main author (KP) screened all titles and abstracts, while two reviewers (BP and MS) independently screened the titles and abstracts of 50% of retrieved articles. Disagreements between the reviewers were resolved through discussion. The same three reviewers (KP, BP, MK) independently reviewed the full texts of the remaining eligible publications and disagreements that occurred were resolved by discussion within the team.

### 2.4. Data extraction

The combined data extraction tool to capture both the quantitative and qualitative findings from the included studies was developed by KP, FZ and LA, following the JBI-MAStARI (Joanna Briggs Institute Meta-Analysis Of Statistics Assessment And Review Instrument) and JBI-QARI (Joanna Briggs Institute Qualitative Assessment And Review Instrument) tools suggested by the JBI reviewers manual (23). Data extraction for the included studies was completed by KP and verified by two other reviewers (BP, MK). The following data were extracted: study reference information (author, year, country), study design, study setting, sample sizes (children and parents/carers), children and parents/carers characteristics (sex, age, and socio-economic status), intervention characteristics (content/components, frequency and duration), quantitative outcomes (e.g., plaque index, knowledge score, behaviour change) and/or qualitative outcomes (e.g., experience, attitude and beliefs of parents and caregivers towards using mobile phone applications).

### 2.5. Risk of bias (quality) assessment

Given the diverse study designs included in this review, quality assessment was undertaken using the Quality Appraisal for Diverse Studies (QuADS) (27). The QuADS quality appraisal tool evaluates key domains such as the underlying theory, defined objectives, appropriateness and rigour of design, data collection methods, and analytical methods. This tool composes of 13 evaluative indicators, with each indicator measured on a four-point Likert scale, from 0 (not at all) to 3 (complete) to enable researchers to distinguish the degree to which a criterion is met. The QuADS criteria have been shown to have strong content and face validity as well as inter-rater reliability (27). The quality assessment tool was piloted with 10% of the sample, by KP, LA and FZ. The main author (KP) and the co-reviewers (LA, FZ) independently applied the QuADS tool to access the quality of the included articles. Discrepancies and final scores were finalised through discussions.

However, the QuADS does not assess the critical risk of bias for quantitative studies such as random sequence generation, allocation concealment and blinding of participants and personnel or outcomes. Therefore, we have performed a further risk assessment of quantitative and mixed methods studies to explore and synthesise these risks of bias.

### 2.6. Data synthesis

Data synthesis was performed using a convergent segregated approach as outlined in the Joanna Briggs Institute mixed-methods review manual (23). A configurative analysis was used to integrate the quantitative and qualitative evidence, where the findings were set side by side with each other, and a narrative description of the findings was made to allow integration of the quantitative and qualitative findings. However, it was not possible to combine results in the form of a quantitative meta-analytic synthesis due to great heterogeneity across the reviews arising from differences in sample characteristics, as well as the differences between the methods present in the primary studies they incorporated. Findings from the qualitative study were narratively synthesised. To provide measures of variance in quantitative findings, confidence intervals (CI) were calculated for studies in which mean and standard deviation (SD) were reported (28).

## 3. Results

**Figure 1:**
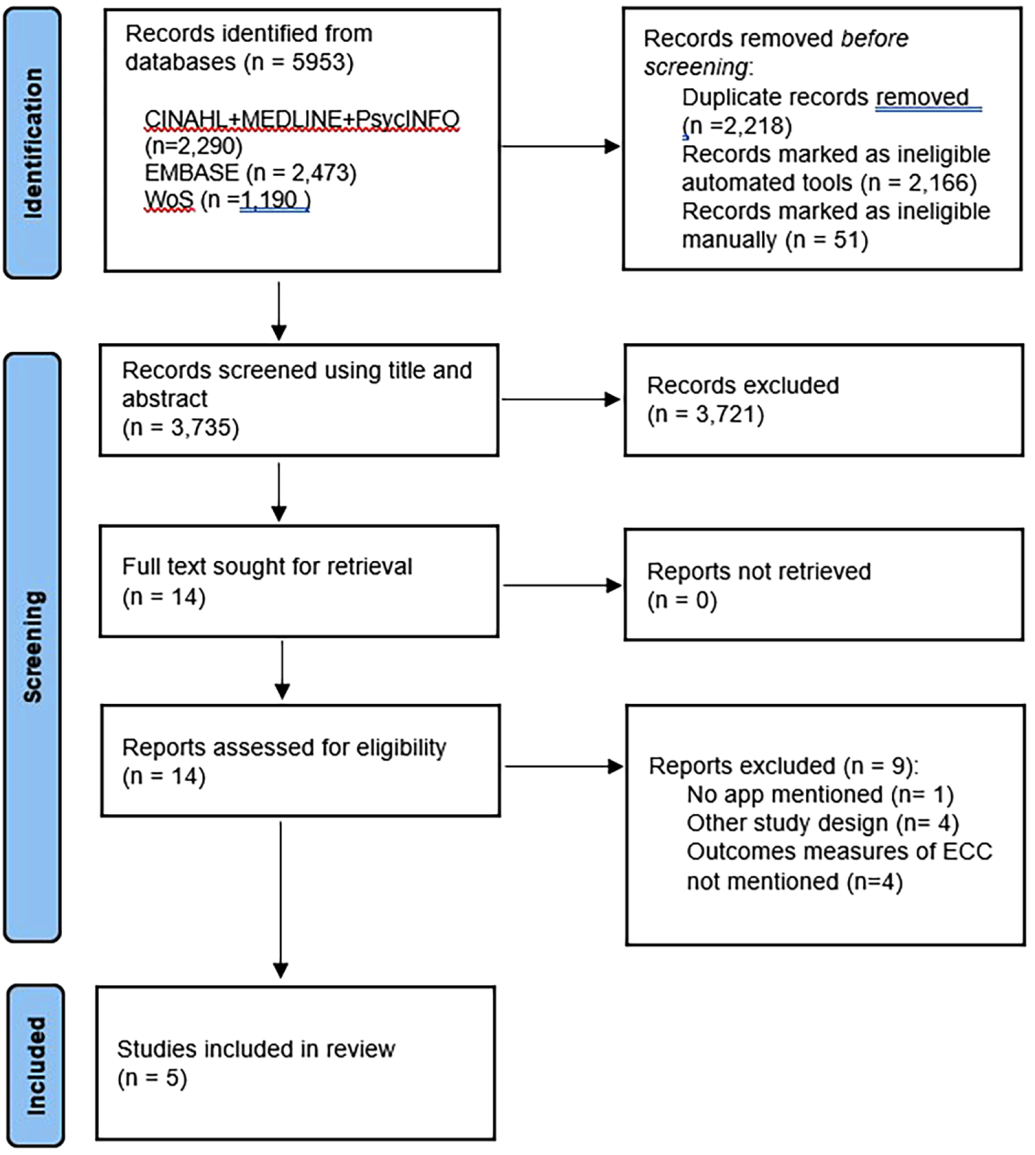
PRISMA flow diagram.

The PRISMA flowchart shown in Figure 1 presents the search results. A comprehensive search of the selected databases retrieved 5,953 records. of which five met the inclusion criteria and were included in this review (shown in Figure 1).

### 3.1. Descriptive synthesis

Out of the five included studies, there was one RCT (22), two before and after studies (21, 29), and two mixed-method studies (30, 31). The total number of participants across the included studies was 4,218. The RCT (22) included children aged 5–6 years in which questionnaires about the current oral hygiene behaviour of children were completed by their parents. The remaining four studies (21, 29–31) focused on parents and caregivers of children aged 0–6 years of age. Only one study (22) included a control group. The RCT (22) was carried out in Germany, the two before and after studies (21, 29) in Saudi Arabia and the two mixed-method studies (30, 31) in the United States. A summary of the included studies is given in Table 1 and the detailed characteristics of the included studies are presented in Supplementary File S2.

**Table 1:** Summary of included five studies.

Author (year)	Country	Study design	Study setting	Sample size	Intervention components	Findings
Alkilzy et al. (22)	Germany	Randomised controlled trial	Paediatric dentistry practice	60	Manual toothbrush Rainbow Vigilant, with a digital motion 3D sensor system (gyroscope), follows toothbrushing movements which are sent to a smartphone (Android/iOS) *via* Bluetooth	Improved medium-term oral hygiene.
Alklayb et al. (29)	Saudi Arabia	Before and after study	Paediatric primary care centre	3,879	An app (iTeethey™) provided information about oral health care for children from infancy to 6 years of age and expecting mothers.	Improved mothers’ knowledge of their children's dental health.
Alqarni et al. (21)	Saudi Arabia	Before and after study	Not stated	230	An app (Your Child Smile) to inform parents on a child's dental health prepartum and from infancy to adolescence	Improved parents’ knowledge of their children's dental health. Apps are effective.
Nolen et al. (31)	USA	Mixed method study	App user testing service	8	A smartphone application prototype, ToothSense, is an oral health promotion tool for the prevention of Early Childhood Caries based on the Theory of Planned Behaviour to aid parents to make good oral health habits part of their pre-schooler's daily routine.	Highlighted the importance and usability of smartphone app for oral health promotion.
Lozoya et al. (30)	USA	Two-phase, sequential embedded mixed methods research design	Public preschools and local medical and dental offices at the Rio Grande	41	Same as above, ToothSense	No effect on intention and behaviour, but social norms and perceived behavioural control emerged as significant predictors of intentions and behaviour.

### 3.2. Characteristics of the interventions

#### 3.2.1. Intervention aims

Of the five studies, the RCT (22) investigated the effectiveness of using a manual toothbrush that is supplied with a gravity sensor and a mobile toothbrushing app among 5 to 6-year-old children. Both the before and after studies (21, 29) compared the effectiveness of mobile applications *iTeethey*™ *and Your Child Smile* respectively on improving children's oral health knowledge among parents. The 2019 mixed-method study (30) pilot tested the *Toothsense* app to explore the influence of smartphone apps on parents’ attitudes, subjective norms (SN), intentions and perceived behaviour control (PBC) of the oral health behaviours of their preschool children. The other mixed-method study (31) developed an app prototype based on the Theory of Planned Behaviour (TPB) and also tested the application for acceptance among the target audience.

#### 3.2.2. Intervention components and outcomes assessed

The RCT (22) ran for 3 months, with data collected at baseline, 6 weeks, and 12 weeks. The study measured the mean and standard deviation of the oral health indices; Quigley-Hein plaque index (QHI) and papillary bleeding index (PBI). The before and after studies (21, 29) ran over different timescales; one (21) for 3 months and the other (29) for 15 days.

One of the two before and after studies provided information about oral health care for children from infancy to 6 years of age and expecting mothers (29), whereas the other provided information to parents on child's dental health from prepartum and infancy to adolescence (21). The information available in these apps covered topics such as pregnancy and dental health, child's teeth, dental disease prevention, diet and children's dental health. The study by AlKlay and co-workers (29) checked for the improvement in the knowledge score of the mothers, and the other study (21) assessed knowledge about tooth development, the importance of deciduous teeth, the importance of regular dental check-ups, pit and fissure sealants, bedtime bottle use, and consequence of early loss of deciduous teeth. Both these studies lacked detailed information on the structure and components of the mobile applications they employed. The two mixed-method studies (30, 31) described the same oral health promotion app for the prevention of ECC called the *Toothsense*, which was developed and beta-tested by the authors. One of the mixed-method studies (30) explored how the *Toothsense* app influenced the attitudes, beliefs, PBC, and intentions of parents of pre-schoolers through a 4-week intervention. In the study by Lozoya et al. (30), the participants received an email with instructions to download the smartphone app and set up push notifications for brushing reminders. Following confirmation of successful app installation, participants were emailed to complete the pre-intervention questionnaire, and they were instructed to use the app twice daily for 4 consecutive weeks before completing a post-intervention questionnaire.

Only two of the included studies (29, 31) stated that the app's content on oral-health information (OHI) was obtained from the American Dental Association (ADA) website. In both studies, participants completed two questionnaires to test their knowledge of their child's oral health at initial registration and following application use over the course.

### 3.3. Quality of studies

All included papers were rated for quality using the QuADS criteria (27). Three articles met the criterion “theoretical or conceptual underpinnings of the research”, which describes the ideas or concepts that frame the study, as well as how the work done is evident in the design, materials, and outcomes discussed. Only two studies (22, 29) received a two-point score for meeting the criterion “appropriate sampling to address the research aim/s” since they did not provide any detailed evidence of whether or not they employed any iterative sampling method in relation to the research objectives or case chosen for study. None of the studies fully engaged stakeholders in the research design provided clear descriptions of the research setting and target population or discussed the strengths and limitations of all aspects of the study. The quality assessment results highlight the lack of transparency regarding the research design and methods quality. As the QuADS tool does not provide any formal cut-off to classify the articles into good, fair and poor and leaves it at the discretion of the researcher, in this systematic review, all the studies screened are included despite the scoring they have received.

Given the QuADS limitation on assessment of confounding factors, quantitative and mixed methods studies were further examined for random sequence generation, allocation concealment and blinding of participants and outcome assessor. Alkilzy et al. (22) was the only study which included a controlled group and participants were randomised using a random list. No information was provided in this study concerning allocation concealment. None of the quantitative or mixed methods studies (21, 29–31) have blinded the participants, they were aware of using/testing the app as part of the intervention. Likewise, only the RCT by Alkilzy et al. (22) blinded the outcome assessor (i.e., single-blinded study).

Detailed scoring of the included studies is provided in Supplementary File S3.

### 3.4. Outcome synthesis

The results demonstrating the effectiveness of interventions used in the included studies are presented in Supplementary File S4.

#### 3.4.1. Quantitative findings

##### 3.4.1.1. Clinical outcomes

The only included RCT in this review (22) found statistically significant improvements in oral health indices (plaque and gingival indices; PBI and QHI) in the test group that used a toothbrushing smartphone app with a gaming component compared with the controls at 6-week recall (QHI: *p* < 0.001, 95% CI [0.92, 1.67]; PBI: *p* < 0.00195% CI [0.14, 0.21]) as well as 12-week recall (QHI: *p* < 0.001, 95% CI [0.71, 1.38]; PBI: *p* < 0.001, 95% CI [0.07, 0.24]).

##### 3.4.1.2. Participants' knowledge

According to the two before and after studies (21, 29), mobile-based applications could be an effective tool for educating parents of children under the age of 6 years about their child's oral health and preventive dental care. The study by Alqarni et al. (21) reported a statistically significant (*p* < 0.05) improvement in the knowledge of parents, who downloaded the app, on tooth development, the importance of deciduous teeth, the importance of regular dental check-ups, pit and fissure sealants, bedtime bottle use, and consequence of early loss of deciduous teeth. Likewise, this study found no significant correlation between the age of the mother and the enhancement of the mother's oral health knowledge. The study by AlKlayb et al. (29) revealed a significant improvement (*p* < 0.001, 95% CI [7.0, 7.19]) in maternal oral health knowledge of their child 3 months after the use of the application. It was observed that the knowledge of mothers in Riyadh (urban) has improved more than that of mothers in Najran (rural). The study also reported a significant correlation between regions (*p* = 0.003) and the number of children (*p* = 0.037) in the family on the knowledge score of the mothers. However, the occupation and age of the mother as well as family income had no significant influence on the oral knowledge of the mothers.

While the study by Alkilzy et al. (22), found that utilising a smartphone app with personalised oral health advice and automatic coaching can successfully lower plaque and bleeding indices, it did not offer any insight or data on the improvement of oral health knowledge among parents and caregivers.

##### 3.4.1.3. App usability testing

Nolen et al. (31) developed and tested the app “*ToothSense*” with eight beta testers who rated the app's usability statements using a 5-point Likert scale questionnaire. Study participants on average agreed that the app met three of the five usability criteria. The most common usability concern was regarding the interface design of the OHI and Sugar Bug Status screens, commonly described by the testers as crowded and difficult to read. Testers requested brighter colours and a simpler layout to improve the readability and decrease the number of icons. All participants agreed that the app offered knowledge about oral health, features/information that addressed the risks and benefits of engaging (or not) in oral health behaviours, and skills necessary to uphold children's positive tooth brushing habits. The prototype received positive reviews in terms of concept, the information provided, and the likelihood of recommending the app.

##### 3.4.1.4. Oral health behaviour

The mixed-method study by Lozoya et al. (30) evaluated the effect of the app “*ToothSense*” developed by Nolen et al. (31) on oral health behaviour of preschool children. This study found no effect of the intervention on parents’ behaviour intentions or oral health behaviours including dietary habits, oral health practices, and dental attendance. However, this study showed social norms as significant predictors of dietary behaviours and oral hygiene intentions.

#### 3.4.2. Qualitative findings

The qualitative component of one of the mixed-method studies (31) included video and audio recordings interviews regarding the usability of the app prototype. The following six themes were deduced: (1) interface design, (2) navigation, (3) feedback, (4) terminology, (5) information and (6) health promotion. Participants found the app helpful, especially with regards to the timer, alarm function and OHI information available on the app prototype, indicating that this was new information that helped them understand the risks related to not caring for their child's teeth. However, comments regarding interface design, particularly regarding the colour, images and the location and size of the icons on the screen, received negative feedback. Navigation presented some difficulties for testers, and terminology was another theme they had concerns over not being able to understand the meanings of some buttons.

The study by Lozoya and co-workers (30) consisted of qualitative interviews with a purposive sample of parents from Phase I and resulted in five emergent themes organised across the TPB constructs for attitude, SN, intentions and PBC and smartphone oral hygiene applications. The thematic analysis revealed that parents’ belief in the importance of establishing oral health habits and brushing reminders and videos delivered *via* a mobile application supported efforts to form oral health habits.

## 4. Discussion

To the best of our knowledge, this study is the first systematic review that explored the effectiveness and evidence of mobile health apps aimed at parents and caregivers of children aged 0–6 years that address early childhood caries. The key question of the present review was what types of mobile health applications are available to parents and carers to reduce the risk factors associated with ECC and to review whether there was evidence that these apps were effective. Only five studies met the inclusion criteria, meaning that there are not many studies about mobile apps available to support the target audience of parents and carers of young children are available. To back up this assertion, a systematic search in the Apps store and Google store (18) also identified a lack of apps targeted at parents of children that adequately addressed prevention behaviours associated with fluoride usage and low-cariogenic diets for children aged younger than 6 years.

The results from this systematic review indicate that there is some evidence to suggest that mobile applications could be an effective oral health promotion tool for the prevention of ECC (21, 29–31). Another finding of this systematic review is that parents’ understanding of their child's tooth development, the effects of tooth loss, and the importance of maintaining proper dental hygiene may improve as a result of using oral health apps (21, 29). Social norms, such as approval from the child's paediatrician and family dentist, were found to be a powerful determinant of dietary and dental hygiene intentions. Studies by (21), and (21) observed a significant improvement in the maternal knowledge of children's oral health observed after using the oral health app. These findings are consistent with other research which found that inadequate oral health literacy of parents is significantly related to higher caries incidence in their children and oral health behaviour (14, 32, 33). According to Alqarni et al. (21), the fact that their mobile application was downloaded by 230 parents within the first 15 days of its release indicates that there is a significant level of internet access among parents and a strong interest in understanding the importance of oral health for their children.

Despite the great importance of oral health in children and its positive effects on their future health, very limited applications have been developed in this area. The qualitative findings from the included studies (30, 31) in this review highlighted that the design of the apps is of foremost importance in their effectiveness, and the users felt these apps must consider theories such as perceived behaviours while delivering oral-health messages. These findings also show that the users’ perception of interface design specifically in relation to the colour, images, location and size of the icons on the screen, as well as the app's components such as the usability of navigational elements and use of well-defined terminology to increase clarity and reduce ambiguity, are important. The usability of a mobile app is directly related to its design and ease of use (34). It is essential for a successful oral-health mobile app that aims to bring a behaviour change to be designed in a user-friendly manner. This brings about a connection between the user and the designer, directly resulting in a positive behaviour change (35).

A meta-analysis of 64 studies (36) concluded that the mobile health apps in which a gamification design was included to promote health, using behaviour change techniques, were more successful than the ones without gamification. This meta-analysis focussed on studies that investigated broader health outcomes including oral health, inferring that the use of apps with gamification could be applied to improving the oral health of different populations across various age groups. The use of health promotion theories embedded in the design of mobile apps might bring in the intended health behaviour changes in the user's health behaviour planned (37, 38). While mHealth applications have the potential to enhance knowledge, attitudes, and practices with regard to ECC, it is imperative for oral health professionals to utilise validated assessment tools, such as the Mobile Application Rating Scale (MARS) (39) or the user version of the scale (40–43) to evaluate their engagement, functionality, design, and information accuracy other than user experience, in order to ensure that patients receive accurate information.

Before designing any intervention, another fundamental practice that can be performed is to identify user expectations through a co-design process. This is also relevant while designing mobile oral health apps. A need to co-design and develop apps that address a broad range of modifiable risk factors associated with ECC targeted at parents has also been addressed in a recent systematic review (18). The authors of the latter review also suggest ensuring the highest quality while designing an oral health app the co-design process should include the clinician, researcher, and patient perspectives on evidence-based information and engaging features. The findings of the systematic review by Chen and co-workers (18) also highlight the advantages of involving professionals from the dental field such as dental health commissioners, dental therapists, and hygienists. Future research must broaden its scope and explore various techniques that will help design a successful and sustainable mobile oral health app. End users’ perceptions of the contents and usability of any such app are invaluable to its acceptance among users.

Finally, while mHealth apps offer new opportunities for healthcare professionals, but privacy concerns and low technical literacy hinder their implementation. A socio-ethical perspective and stakeholder participation are necessary for mHealth app development to ensure responsible implementation (44). A major ethical concern surrounding mHealth apps pertains to the amount of data collected and the utilisation of such data by app manufacturers. The absence of proper regulations increases the misuse of data for non-health-related purposes, such as targeted advertising or data mining, and the possibility of personal health information being disclosed or transferred to third parties without the consent of the user. The ethical issues in mHealth are complex and require a pragmatic approach to minimise ethical risk by switching from a privacy-centred frame to a consent-centred frame (45). Creating a high-quality consent process for sensitive material is challenging, but necessary to realise the potential benefits of mHealth.

## 5. Conclusion

This review demonstrated that oral health promotion programs delivered through mobile apps to parents or caregivers with children under the age of 6 years could be effective in improving child oral health awareness among parents and caregivers. However, this review also highlighted the very limited number of interventions on the usage of oral-health mobile applications for parents and caregivers. There is a need for more high-quality studies with a larger number of participants to find out which features of mHealth interventions with parents and carers could effectively be employed to reduce the prevalence of ECC. Further studies and apps should be developed based on evidence-based behaviour change techniques and incorporate features such as gamification to make apps more effective and engaging with our target population.

## Data Availability

The original contributions presented in the study are included in the article/**Supplementary Materials**, further inquiries can be directed to the corresponding authors.
