# Supplementary material for: App-based oral health promotion interventions on modifiable risk factors associated with early childhood caries: A systematic review
Source: Front Oral Health. 2023 Mar 10;4:1125070. doi: 10.3389/froh.2023.1125070 (PMC10036826; doi:10.3389/froh.2023.1125070)
Supplement: Supplementary file 1 [file Table1.docx]

**Supplementary File 1 - Search Strategy**

**Search run across CINAHL, MEDLINE, PsycINFO via EBSCO Host**

| # | Query | Results |
| --- | --- | --- |
| S1 | Mothers OR Maternal OR Mom OR Mum OR carer* OR Pregnan* mother OR Prenatal OR pre natal OR Postnatal OR post natal OR antenatal OR ante natal OR Father OR Dad OR Paternal OR Parent* OR Caregivers OR care giver OR Family | 3,913,279 |
| S2 | Child***** OR Newborn OR Baby OR Babies OR Infant OR Pediatric OR Paediatric | 5,738,992 |
| S3 | S1 OR S2 | 8,043,428 |
| S4 | App OR Mobile application OR mobile phone OR cell* phone OR Smartphone OR smart phone OR iPhone OR i-phone OR iOS OR android OR mobile health OR mHealth OR m-Health OR Tablet OR Handheld computer OR hand-held computer OR Telehealth OR tele-health OR Gamifi* OR Game app | 235,119 |
| S5 | Caries OR ECC OR Tooth decay OR Dental decay OR Carious lesions OR Oral hygiene OR fluorid* OR sweetened beverage OR Free sugar OR Sugar* OR sugary drinks OR tooth brushing OR toothbrushing OR Dietary habits OR Diet* | 1,459,356 |
| S6 | S3 AND S4 AND S5 (Limiters - Published Date: 19970101-20210631; English Language) | 2,290 |

**Search run across EMBASE via Ovid SP**

| # | Query | Results |
| --- | --- | --- |
| 1 | (Mother*1 or Maternal or Mom or Mum or carer* or Pregnan* or Prenatal or pre natal or Postnatal or post natal or antenatal or ante natal or Father*1 or Dad* or Paternal or Parent* or Caregiver* or care giver* or Family).mp. | 2901348 |
| 2 | (Child* or Newborn* or Baby or Babies or Infant* or Pediatric*1 or Paediatric*1).mp. | 2798117 |
| 3 | 1 or 2 | 4855595 |
| 4 | (App*1 or Mobile application* or mobile phone* or cell* phone* or Smartphone* or smart phone* or iphone* or i-phone* or ios or android or mobile health or mHealth or m-Health or Tablet* or Handheld computer*1 or hand-held computer*1 or Telehealth or tele-health or Gamifi* or Game app*1).mp. | 214423 |
| 5 | (Caries or ECC or Tooth decay or Dental decay or Carious lesion* or Oral hygiene or fluorid* or sweetened beverage* or Free sugar or Sugar* or sugary drink*1 or tooth brushing or toothbrushing or Diet* or Dietary habit*1).mp. | 1032789 |
| 6 | 3 and 4 and 5 | 2473 |

**Search run across Web of science (WoS)**

| Set | Query | Results |
| --- | --- | --- |
| # 6 | #5 AND #4 AND #3  Indexes=SCI-EXPANDED, SSCI, A&HCI, CPCI-S, CPCI-SSH, ESCI Timespan=All years | 1,190 |
| # 5 | TOPIC: (Caries OR ECC OR "Tooth decay" OR "Dental decay" OR "Carious lesion*" OR "Oral hygiene" OR fluorid* OR "sweetened beverage*" OR "Free sugar" OR Sugar* OR "sugary drink*" OR "tooth brushing" OR toothbrushing OR Diet* OR "Dietary habit")  Indexes=SCI-EXPANDED, SSCI, A&HCI, CPCI-S, CPCI-SSH, ESCI Timespan=All years | [1,265,356](http://apps.webofknowledge.com/summary.do?product=WOS&doc=1&qid=11&SID=D18PyyovVBHLXgavbq8&search_mode=GeneralSearch&update_back2search_link_param=yes) |
| # 4 | TOPIC: (App OR "Mobile application*" OR "mobile phone*" OR "cell* phone*" OR Smartphone OR "smart phone*" OR iphone OR "i-phone*" OR ios OR android OR "mobile health" OR mHealth OR "m-Health" OR Tablet OR "Handheld computer*" OR "hand-held computer*" OR Telehealth OR tele-health OR Gamifi* OR "Game app*")  Indexes=SCI-EXPANDED, SSCI, A&HCI, CPCI-S, CPCI-SSH, ESCI Timespan=All years | [258,584](http://apps.webofknowledge.com/summary.do?product=WOS&doc=1&qid=8&SID=D18PyyovVBHLXgavbq8&search_mode=GeneralSearch&update_back2search_link_param=yes) |
| # 3 | #2 AND #1  Indexes=SCI-EXPANDED, SSCI, A&HCI, CPCI-S, CPCI-SSH, ESCI Timespan=All years | [4,916,641](http://apps.webofknowledge.com/summary.do?product=WOS&doc=1&qid=7&SID=D18PyyovVBHLXgavbq8&search_mode=CombineSearches&update_back2search_link_param=yes) |
| # 2 | TOPIC: (Child* OR Newborn OR Baby OR Babies OR Infant OR Pediatric OR Paediatric)  Indexes=SCI-EXPANDED, SSCI, A&HCI, CPCI-S, CPCI-SSH, ESCI Timespan=All years | [2,455,556](http://apps.webofknowledge.com/summary.do?product=WOS&doc=1&qid=6&SID=D18PyyovVBHLXgavbq8&search_mode=GeneralSearch&update_back2search_link_param=yes) |
| # 1 | TOPIC: (Mothers OR Maternal OR Mom OR Mum OR carer* OR Pregnan* OR Prenatal OR "pre natal" OR Postnatal OR "post natal" OR antenatal OR "ante natal" OR Father OR Dad OR Paternal OR Parent* OR Caregivers OR "care giver*" OR Family)  Indexes=SCI-EXPANDED, SSCI, A&HCI, CPCI-S, CPCI-SSH, ESCI Timespan=All years | [3,115,212](http://apps.webofknowledge.com/summary.do?product=WOS&doc=1&qid=5&SID=D18PyyovVBHLXgavbq8&search_mode=GeneralSearch&update_back2search_link_param=yes) |
