# Supplementary material for: App-based oral health promotion interventions on modifiable risk factors associated with early childhood caries: A systematic review
Source: Front Oral Health. 2023 Mar 10;4:1125070. doi: 10.3389/froh.2023.1125070 (PMC10036826; doi:10.3389/froh.2023.1125070)
Supplement: Supplementary file 2 [file Table2.docx]

**Supplementary File 2 - Characteristics of included studies**

| Author (year), Country | Study design | Study setting, target population and sample size at baseline | Study demographics (age and Socioeconomic status) | Intervention components (name of the app, intervention development, intervention dissemination, intervention content) | Intervention load (frequency, intensity duration) | Statistical analysis |
| --- | --- | --- | --- | --- | --- | --- |
| Alkilzy et al. (2019),  Germany | **Randomised Controlled Trial** | **Setting:**  Clinical setting (paediatric dentistry practice)  **Target population:** children between the age of 5-6 years  **Sample size:** 60  Intervention group: 30  Control Group:30 | **Age of participants: (children):** 5-6 years  **Income/ Educational status of participants:** not recorded | **Name of the app:** not specified  **Intervention development:** not clear  **Intervention dissemination:** Participants were randomly allocated to two groups:  Intervention group (n = 30) received QR code and assistance for the installation and operation of the toothbrush application on a smartphone or tablet. The measured data of the gravitational sensor were then transmitted via Bluetooth to the parent’s smartphone.  Control group (n = 30) received no announcement of the QR code & toothbrushing was achieved with the same manual toothbrush as in the Intervention group but without additional use of the motivational app.  **Intervention content:**  A manual toothbrush Rainbow Vigilant, with a standard, flat bristle surface in an oval multitoothed brushing plane was given to each child. The handle was composed of a digital motion 3D sensor system (gyroscope) so that the toothbrush follows the toothbrushing movements of the child in real-time and was sent via Bluetooth to a smartphone (Android/iOS). Via Smart-Bluetooth and an iOS or Android smartphone, the application analyses the toothbrushing movements. Using the motion sensors, the toothbrush detects the children’s brushing habits This was then compared in real-time via a mobile application. The app saves the data of each brushing session and adds bonus points. The closer the child got to the optimal brushing movements, the more points were awarded. | The study had included a total of 4 visits including a pre-experimental visit to check for eligibility of each participant and to sign the consent forms in paediatric dental practice.  **6-week recall**  Intervention Group: Quigley-Hein plaque index (QHI), papilla bleeding index (PBI), checking the toothbrushing technique, returning the toothbrush and the motivational app, n = 26.  Control Group: QHI, PBI, checking the toothbrushing technique, returning the toothbrush, n = 23.  *6 weeks without special toothbrush or motivational app, using personal toothbrush and oral hygiene products.*  **12-week recall**  Intervention Group: Status of general oral hygiene incl. QHI, PBI, checking the toothbrushing technique, n = 26.  Control Group: Status of general oral hygiene incl. QHI, PBI, checking the toothbrushing technique, n = 23. | The data analysis was performed with SPSS 14.0 for Windows (SPSS Inc., Chicago, IL, USA).  The Friedman test was applied for intragroup analysis and Mann-Whitney U tests for analysis between the two groups.  Descriptive statistics were used to analyse the participant’s oral hygiene. |
| Alklayb et al. (2017), Saudi Arabia | **Before and after study** : | **Setting:**  Clinical setting (paediatric primary care centre)  **Target population** mothers of children aged <71 months (~6 years) of age  **Sample size**: 3879  No control group | **Mean ± SD age of participants (mothers):**   - Riyadh-Urban   28.18 ± 3.45 years   - Najran-Rural   28.05 ± 3.82 years  **Mean ± SD monthly income of mothers:**   - Riyadh-Urban 14,210.23 ± 3561.0 - Najran-Rural 13,333.71 ± 3494.73 | **Name of the app**: iTeethey™.  **Intervention development:**  Developed using guidelines the American Academic Paediatric Dentistry (AAPD,2013).  **Intervention dissemination:** distributed to 3879 mothers of children below 6 years of age (1989 in Riyadh Region and 1890 in Najran region). A total of 1055 mothers downloaded the application.  **Intervention content:** Information about oral health care for children from infancy to 6 years of age &expecting mothers. Available in English and Arabic and hosted on the App Store. | Mothers completed a previously validated Arabic tool designed to test their knowledge of their child’s oral health& followed up for usage statistics using the online data usage statistics (http:// applicius.com/).  Reminders were sent after 3 months to those mothers who had used the application at least once in the past 3 months  Those responded completed the same validated tool in Arabic. | Overall impact of the mobile application was evaluated using the paired sample *t*-test.  A Chi-square test was employed to compare the follow-up rate between the two study sites. |
| Alqarni et al. (2018)  Saudi Arabia | **Before and after study** : | **Setting**: not mentioned  **Target population** parents of children from prenatal health up to 15 years of age  **Sample** **size**: 230  No control group | **Age of participants (parents):** not recorded  **Income/ Educational status of participants**: not recorded | **Name of the app**: Your Child Smile  **Intervention development:** no information provided  **Intervention dissemination:** The app was developed and made accessible on the app store and the play store.  **Intervention content:** Provided information to parents on child’s dental health prepartum and from infancy to adolescence covering topics such as; pregnancy and dental health, your child’s teeth, dental disease prevention, diet and children’s dental health. The application also provided interactive answers to parents’ questions regarding information that parents may want to ask from specialists concerned with the treatment of infants, children, and adolescents. | Parents completed two similar questionnaires at initial registration and following application use throughout the 15-day research period, with 110 answering only the first and 120 answering both. | The responses were entered into a Microsoft Excel spreadsheet and analysed with the International Business Machines Statistical Package for the Social Sciences (SPSS) version 20 and descriptive analysis was performed. |
| Nolen et al (2018)  USA | **Mixed method study** | **Setting**: A user testing service was used to test the app (User Testing; Mountain View, CA),  **Target population** parent or primary caregiver of one or more children age 6 years or younger  **Sample** **size**: 8 beta 4testers, 6 females and 2 males  No control group | **Age of participants:**  22 to 36 years with an average age of 27.5 years  **Income/educational status of participants:**  <40k : 1 participant  40-100k: 7 participants | **Name of the app**: Toothsense  **Intervention development:**  The prototype design and development of the app were based on the Theory of Planned Behaviour (TPB). The Behavioural Intervention Technology (BIT) model was used to ensure the development process was replicable and systematic. An interdisciplinary team including a dental hygienist (primary researcher), graphic motion designer, and two app software developers collaborated to develop and beta test this oral health promotion prototype app. All oral health information (OHI) was obtained from the American Dental Association (ADA) website.  **Intervention dissemination:** Testers were presented with a series of tasks and prompts followed by a 5-point Likert-scale questionnaire that quantitatively measured perceptions of the app’s interactive design based on Jakob Nielsen’s principles and behavioural strategies. A camera was placed on the shoulder of each beta tester while they conducted the tasks to record the video and audio and they were encouraged to think aloud and talk through their actions and feelings.  **Intervention content:** The app prototype features included goal setting, motivation, monitoring, and feedback to account for Doshi’s Intervention Strategies (DIS) by Behaviour Change Model or Theory. The app prototype features for goal setting were found in the settings screen. The app prototype accounted for monitoring and feedback on the following features: Sugar Bug Status, Brush Along, Smiles Club, and Mouth Journal. Interactive features incorporated into the prototype app included information delivery, notifications, logs, peer support, reviews, and visualisation. | The beta testers used the prototype app for an average of 15 minutes. | Responses were recorded using a 5-point Likert item (1=strongly disagree, 2=disagree, 3=neither agree or disagree, 4=agree, 5=strongly agree) and were tallied to obtain a mean score for each question on usability and perceptions of TPB.  A template approach using codes was applied to organise data from audio and video recordings & Preliminary codes were developed based on the recordings and the theoretical framework of TPB. Analysis of texts was guided by these preliminary codes |
| Lozoya et al. (2019), USA | **two-phase,**  **sequential embedded mixed methods research design** | **Setting**: public preschools and local medical and dental offices at the Rio Grande  **Target population** parents of at least one pre-schooler with a mean (3.48 ± 0.93) years who use an iPhone.  **Sample size:** 41  No control group | **Age of participants:** not recorded  **Income/ Educational status of participants:**  *Highest education completed by mother*  Pre-post intervention frequency, n=33(%),n=26(%)  Other 1(3%), 1(3%)  college/associate degree 14(42.4%), 12(36.4%)  Bachelor degree 8(24.2%), 8(24.2%)  Graduate/post-graduate degree 8(24.2%), 5(15.2%)  *Highest education completed by father*  Pre-post intervention frequency, n=33(%),n=26(%)  High school 10(30.3%), 8(24.2%) college/associate degree 8(24.2%), 8(24.2%)  Bachelor degree 8(24.2%), 6(18.2%)  Graduate/post-graduate degree 5(15.2%) 4(12.1%) | **Name of the app**: Toothsense  **Intervention development:**  **Phase I**: A 124-item validated questionnaire designed by Van den Branden et al. to measure oral health behaviours in children and Theory of Planned Behaviour (TPB) determinants were used.  **Phase II**: the semi-structured interviews using 13 open-ended questions were based on previous research by Zoellner et al  **Intervention dissemination:**  Participants received an email with instructions to download the smartphone app and create push notifications for brushing reminders, which also served as a reminder to use the app daily. After confirmation of the successful app installation, participants received an email to take the pre-intervention questionnaire using a web-based survey tool.  Out of 41 parents, 33 participants completed the pre-intervention questionnaire. Participants were instructed to use the app twice a day for four consecutive weeks.  The post-intervention questionnaire was completed by 26 participants. They were also given an incentive and also information on phase two.  Phase II included semi-structured interviews with 13 open-ended questions. Participants who completed the interview received an additional incentive.  **Intervention content:**  **Phase I:** The questionnaire consisted of 71 belief-based items related to three primary scales: dietary habits (24 items), oral hygiene (22 items), and dental attendance (25 items). The belief-based items included questions regarding attitude, intention, Subjective Norms (SN), self-reported behaviour and Perceived Behavioural Control (PBC). There were additional questions to gather background information on dental care (14 items), children’s oral hygiene habits (7 items), nutrition (17 items), parents’ oral health (8 items), and demographics (4 items).    **Phase II:** In the interview, the participants were asked questions on how they cared for their children’s oral health based on the TPB determinants including attitude, SN, intentions, and PBC following the use of the smartphone app. Responses were gathered using virtual interviews recorded and conducted using a video conferencing platform. | Phases I and II took place over four weeks. | Differences in behavioural intention and reported behaviour which were collected through semi-structured interviews were examined using a group t-test to compare the pre and post-intervention.  Linear regression analysis was used to determine the predictive relationship of each TPB subscale on the domain matching intention and behaviour |
