# Supplementary material for: App-based oral health promotion interventions on modifiable risk factors associated with early childhood caries: A systematic review
Source: Front Oral Health. 2023 Mar 10;4:1125070. doi: 10.3389/froh.2023.1125070 (PMC10036826; doi:10.3389/froh.2023.1125070)
Supplement: Supplementary file 3 [file Table3.docx]

**Supplementary File 3 - QuADS Quality Appraisal**

| Study ID | Theoretical or conceptual underpinning to the research | Statement of research aim/s | Clear description of research  setting and target population | The study design is  appropriate to address the stated  research aim/s | Appropriate sampling to  address the research aim/s | Rationale for choice of data  collection tool/s | The format and content of data  collection tool is appropriate to  address the stated research  aim/s | Description of data collection procedure | Recruitment data provided | Justification for analytic  method selected | The method of analysis was  appropriate to answer the  research aim/s | Evidence that the research  stakeholders have been  considered in research design or  conduct. | Strengths and limitations  critically discussed |
| --- | --- | --- | --- | --- | --- | --- | --- | --- | --- | --- | --- | --- | --- |
| Alkilzy | 2 | 2 | 2 | 3 | 2 | 2 | 3 | 0 | 3 | 0 | 2 | 0 | 1 |
| Alkalyb | 2 | 2 | 3 | 2 | 2 | 2 | 2 | 2 | 3 | 2 | 2 | 0 | 1 |
| Alqarni | 1 | 2 | 1 | 1 | 0 | 0 | 2 | 1 | 1 | 1 | 1 | 0 | 1 |
| Nolen | 3 | 3 | 0 | 1 | 0 | 3 | 2 | 3 | 0 | 0 | 1 | 0 | 2 |
| Santi Lozoya (Pre-intervention) | 1 | 3 | 0 | 2 | 0 | 1 | 2 | 2 | 2 | 2 | 2 | 0 | 2 |

**Note: Coding reference**

1. *Theoretical or conceptual underpinning to the research:*

0 No mention at all

1 General reference to broad theories or concepts that frame the study. e.g. key concepts were identified in the introduction section.

2 Identification of specific theories or concepts that frame the study and how these informed the work undertaken. e.g. key concepts were identified in the introduction section and applied to the study.

3 Explicit discussion of the theories or concepts that inform the study, with application of the theory or concept evident through the design, materials and outcomes explored. e.g. key concepts were identified in the introduction section and the application apparent in each element of the study design.

1. *Statement of research aim/s*

0 No mention at all

1 Reference to what the sought to achieve embedded within the report but no explicit aims statement.

2 Aims statement made but may only appear in the abstract or be lacking detail.

3 Explicit and detailed statement of aim/s in the main body of report.

1. *Clear description of research setting and target population*

0 No mention at all.

1 General description of research area but not of the specific research environment e.g. ‘in primary care.’

2 Description of research setting is made but is lacking detail e.g. ‘in primary care practices in region [x]’.

3 Specific description of the research setting and target population of study e.g. ‘nurses and doctors from GP practices in [x] part of [x] city in [x] country.’

1. *The study design is appropriate to address the stated research aim/s*

0 No research aim/s stated or the design is entirely unsuitable e.g. a Y/N item survey for a study seeking to undertake exploratory work of lived experiences.

1 The study design can only address some aspects of the stated research aim/s e.g. use of focus groups to capture data regarding the frequency and experience of a disease.

2 The study design can address the stated research aim/s but there is a more suitable alternative that could have been used or used in addition e.g. addition of a qualitative or

3 The study design selected appears to be the most suitable approach to attempt to answer the stated research aim/s. quantitative component could strengthen the design.

1. *Appropriate sampling to address the research aim/s*

0 No mention of the sampling approach.

1 Evidence of consideration of the sample required e.g. the sample characteristics are described and appear appropriate to address the research aim/s.

2 Evidence of consideration of sample required to address the aim. e.g. the sample characteristics are described with reference to the aim/s.

3 Detailed evidence of consideration of the sample required to address the research aim/s. e.g. sample size calculation or discussion of an iterative sampling process with reference to the research aims or the case selected for study.

1. *Rationale for choice of data collection tool/s*

0 No mention of rationale for data collection tool used.

1 Very limited explanation for choice of data collection tool/s. e.g. based on availability of tool.

2 Basic explanation of rationale for choice of data collection tool/s. e.g. based on use in a prior similar study.

3 Detailed explanation of rationale for choice of data collection tool/s. e.g. relevance to the study aim/s, co-designed with the target population or assessments of tool quality.

1. *The format and content of data collection tool is appropriate to address the stated research aim/s*

0 No research aim/s stated and/or data collection tool not detailed.

1 Structure and/or content of tool/s suitable to address some aspects of the research aim/s or to address the aim/s superficially e.g. single item response that is very general or an open-response item to capture content which requires probing.

2 Structure and/or content of tool/s allow for data to be gathered broadly addressing the stated aim/s but could benefit from refinement. e.g. the framing of survey or interview questions are too broad or focused to one element of the research aim/s.

3 Structure and content of tool/s allow for detailed data to be gathered around all relevant issues required to address the stated research aim/s.

1. *Description of* *data collection procedure*

0 No mention of the data collection procedure.

1 Basic and brief outline of data collection procedure e.g. ‘using a questionnaire distributed to staff’.

2 States each stage of data collection procedure but with limited detail or states some stages in detail but omits others e.g. the recruitment process is mentioned but lacks important details.

3 Detailed description of each stage of the data collection procedure, including when, where and how data was gathered such that the procedure could be replicated.

1. *Recruitment data provided*

0 No mention of recruitment data.

1 Minimal and basic recruitment data e.g. number of people invited who agreed to take part.

2 Some recruitment data but not a complete account e.g. number of people who were invited and agreed.

3 Complete data allowing for full picture of recruitment outcomes e.g. number of people approached, recruited, and who completed with attrition data explained where relevant.

1. *Justification for analytic method selected*

0 No mention of the rationale for the analytic method chosen.

1 Very limited justification for choice of analytic method selected. e.g. previous use by the research team.

2 Basic justification for choice of analytic method selected e.g. method used in prior similar research.

3 Detailed justification for choice of analytic method selected e.g. relevance to the study aim/s or comment around of the strengths of the method selected.

1. *The method of analysis was appropriate to answer the research aim/s*

0 No mention at all.

1 Method of analysis can only address the research aim/s basically or broadly.

2 Method of analysis can address the research aim/s but there is a more suitable alternative that could have been used or used in addition to offer a stronger analysis.

3 Method of analysis selected is the most suitable approach to attempt answer the research aim/s in detail e.g. for qualitative interpretative phenomenological analysis might be considered preferable for experiences vs. content analysis to elicit frequency of occurrence of events.

1. *Evidence that the research stakeholders have been considered in research design or conduct.*

0 No mention at all.

1 Consideration of some the research stakeholders e.g. use of pilot study with target sample but no stakeholder involvement in planning stages of study design

2 Evidence of stakeholder input informing the research. e.g. use of pilot study with feedback influencing the study design/conduct or reference to a project reference group established to guide the research.

3 Substantial consultation with stakeholders identifiable in planning of study design and in preliminary work e.g. consultation in the conceptualisation of the research, a project advisory group or evidence of stakeholder input informing the work.

1. *Strengths and limitations critically discussed*

0 No mention at all.

1 Very limited mention of strengths and limitations with omissions of many key issues. e.g. one or two strengths/limitations mentioned with limited detail.

2 Discussion of some of the key strengths and weaknesses of the study but not complete. e.g. several strengths/limitations explored but with notable omissions or lack of depth of explanation.

3 Thorough discussion of strengths and limitations of all aspects of study including design, methods, data collection tools, sample & analytic approach

**A more comprehensive explanation of the quality of the included studies according to the QuADS criteria.**

Note: Justification is provided for scores of ≤2 (i.e., when the criteria do not meet the full score of 3)

**Alkilzy et a.,(2019) :**

This RCT study identified the key concepts and applied them to the study but explicit discussion of the application of the concept was not evident through the design (*Criteria- Theoretical or conceptual underpinning to the research*). The research aims were stated, but only appeared in the abstract and lacked specific detail in the main body of the report (*Criteria - Statement of research aim/s*). The description of the research setting was mentioned in the study, but there were no details regarding the target population (*Criteria - Clear description of research setting and target population*). The study design appeared to be the most suitable approach to attempt to answer the stated research aims, but the addition of a quantitative component would have improved the design (*Criteria - The study design is appropriate to address the stated research aim/s).* The study did not provide adequate evidence for the calculation of sample size or the process for selecting the sample in relation to the research aims (*Criteria - Appropriate sampling to address the research aim/s*). The choice of data collection tool was not explained in detail, including its relevance to the study aims or assessments of tool quality (*Criteria- Description of data collection procedure*). The study did not provide any justification for the selected analytic method (*Criteria- Justification for analytic method selected*). Stakeholder consultation was not referred to at any point during the study's design or its preliminary research (*Criteria- Evidence that the research stakeholders have been considered in research design or conduct*). There was very limited mention of strengths and limitations with omissions of several key issues (*Criteria- Strengths and limitations critically discussed*).

**Alklayb et al. (2017):**

This before-and-after study was supported by a number of theories and concepts, although these were not adequately articulated. Even though certain important principles were mentioned in the introduction, there was no indication of how these concepts would be applied to the study design (*Criteria- Theoretical or conceptual underpinning to the research*). Although the research environment and target audience were well described, the study's objectives were not clearly stated in the report's main body (*Criteria - Statement of research aim/s*). Although the sample size calculation and the iterative sampling process were not sufficiently supported by the study (*Criteria - Appropriate sampling to address the research aim/s*), the study did provide a thorough description of the research setting and the intended audience. The study design was effective in achieving the stated research aims, however adding a quantitative element might have made the design stronger (*Criteria- The study design is appropriate to address the stated research aim/s*). Previous studies with a similar design provided the basis for the selection of the data collection approach, but the tools used to collect the necessary data to meet the research objectives were not sufficiently developed (*Criteria – Rationale for choice of data collection tool/s*). The lack of complete details regarding when, where, and how the data were collected make it difficult to duplicate the data collection process (*Criteria- Description of data collection procedure*). Despite the fact that the data collecting tool employed was successful in obtaining the information it required, the study's content and structure still have room for improvement (*Criteria- The format and content of data collection tool is appropriate to address the stated research aim/s*). The analytical method was chosen solely because it had been used in previous, comparable research; however, this strategy might not be the best one to fully meet the research aims (*Criteria- Justification for analytic method selected*). Evidence of the research stakeholders’ involvements throughout design or implementation was not stated (*Criteria- Evidence that the research stakeholders have been considered in research design or conduct*). This study offered comprehensive data that allowed for a full picture of recruiting outcomes, including the number of participants approached, recruited, and who completed with attrition data explained where pertinent (*Criteria- Recruitment data provided*). There was only a brief mention of one or two of the study's drawbacks (*Criteria- Strengths and limitations critically discussed*).

**Alqarni et al., (2018):**

The key concepts were outlined in the introduction of this before-and-after study, but the theory or concept's applicability was not made clear in the study's design, materials, or outcomes (*Criteria- Theoretical or conceptual underpinning to the research*). In the main body of the study, there was no explicit or comprehensive statement of the study's aims (*Criteria - Statement of research aim/s & Criteria - Appropriate sampling to address the research aim/s*). A general description of the research area was provided (*Criteria - Clear description of research setting and target population*), but neither the study's target population nor its particular research setting was mentioned. The study failed to provide sufficient evidence of considering the sample size required to address the research aims (*Criteria - Appropriate sampling to address the research aim/s*), the justification for choosing the data collection method (*Criteria – Rationale for choice of data collection tool/s*), or stakeholder consultation during the study's preliminary research development or implementation of the study (*Criteria- Evidence that the research stakeholders have been considered in research design or conduct*). The research team's decision to adopt the analytical procedure was not a fully justified method (*Criteria- Justification for analytic method selected*); only that it had been used in the past was mentioned. There was a need for a more thorough justification that included the benefits of the chosen method and how it correlated to the research aims (*Criteria- The method of analysis was appropriate to answer the research aim/s*). Additionally, this research lacked a thorough analysis of the advantages and disadvantages of all study components, including the design, methodologies, data collection tools, sample, and analytical approach (*Criteria- Strengths and limitations critically discussed*).

**Nolen et al., (2018):**

This study did not indicate the target population or provide any recruiting data information (*Criteria - Clear description of research setting and target population*). Furthermore, the study also failed to specify the method of sampling employed for reaching the research objectives (*Criteria - Appropriate sampling to address the research aim/s*). The study design only addressed some aspects of the stated research aims and the framing of interview questions were too broad, focusing only on one element of the research aims (*Criteria- The study design is appropriate to address the stated research aim/s*). The failure to address the research aims adequately and comprehensively indicated the weakness in the research methodology and a lack of detailed analysis (*Criteria- The method of analysis was appropriate to answer the research aim/s*). Certain strengths and limitations were analysed, but with an insufficient explanation of the study (*Criteria- Strengths and limitations critically discussed*).

**Santi et al. (2019)**

The main principles of the study were presented in the introduction section, and the authors used general references to broad theories and ideas to frame the research (*Criteria- Theoretical or conceptual underpinning to the research)*. But it was not demonstrated how these ideas may be applied to each part of the study design (*Criteria- The study design is appropriate to address the stated research aim/s*). The sole justification provided for the selection of the data-gathering tools was that they were chosen based on availability (*Criteria – Rationale for choice of data collection tool/s*). The study failed to provide any explanation for the theoretical basis of the research, and the sampling method used to meet the research aims (*Criteria - Appropriate sampling to address the research aim/s*). No reference was made to the extent of stakeholder involvement in the study design or preliminary work (*Criteria- Evidence that the research stakeholders have been considered in research design or conduct*). The analysis approach chosen was suitable for addressing the research objectives, although there might have been a stronger alternative that could have been included. (*Criteria- The method of analysis was appropriate to answer the research aim/s*). The tools used for data collection allowed for data to be broadly gathered and to meet the research goals but could benefit from improvement (*Criteria- The format and content of data collection tool is appropriate to address the stated research aim/s).* The study described each stage of the data collection process, but with limited detail or missing certain stages (*Criteria- Justification for analytic method selected*). Some recruitment data was provided, but it was not a complete account, for example, it did not include the number of individuals who were invited and agreed to participate (*Criteria- Recruitment data provided*). The choice of the analytical method used was justified with basic reasoning, such as its prior use in similar research (*Criteria- The method of analysis was appropriate to answer the research aim/s*). Only a few of the study's limitations were acknowledged (*Criteria- Strengths and limitations critically discussed*).

.
