# Supplementary material for: App-based oral health promotion interventions on modifiable risk factors associated with early childhood caries: A systematic review
Source: Front Oral Health. 2023 Mar 10;4:1125070. doi: 10.3389/froh.2023.1125070 (PMC10036826; doi:10.3389/froh.2023.1125070)
Supplement: Supplementary file 4 [file Table4.docx]

**Supplement File 4 - Outcomes of the Interventions Used in the Included Studies**

**(Note: CI* - Confidence intervals were calculated from mean and standard deviation reported in each article)**

| Author (year), Country | Outcome Measures | Quantitative outcomes | Qualitative Outcomes | Author’s Conclusion |
| --- | --- | --- | --- | --- |
| Alkilzy et al, (2019),  Germany | (1) Quigley-Hein plaque index (QHI)  (2) papillary bleeding index (PBI) | **Mean ±SD QHI at:**  (1) Baseline  (1a) Test group 2.36±0.74  (1b) Control group 2.42±0.77  (1c) *p* value 0.94  (1d) 95% CI* [-0.21, 0.33]  (2) 6-week recall  (2a) Test group 0.58±0.48  (2b) Control group 1.88±0.88  (2c) *p* value <0.001  (2d) 95% CI* [0.92, 1.67]  (3) 12-week recall  (3a) Test group 0.44±0.48  (3b) Control group 1.49±0.73  (3c) *p* value <0.001  (3d) 95% CI* [0.71, 1.38]  (4) Differences in mean ranks within each group  (4a) χ2 (2, n = 26) = 40.722 p < 0.01  (4b) χ2 (2, n = 23) = 27.573 p < 0.01  **Mean ±SD PBI at:**  (1)Baseline  (1a) Test group 0.42±0.21  (1b) Control group 0.47±0.28  (1c) *p* value 0.59  (1d) 95% CI* [-0.04, 0.14]  (2) 6-week recall  (2a) Test group 0.08±0.13  (2b) Control group 0.26±0.00  (2c) *p* value <0.001  (2d) 95% CI* [0.14, 0.21]  (3) 12-week recall  (3a) Test group 0.05±0.08  (3b) Control group 0.21±0.14  (3c) *p* value <0.001  (3d) 95% CI* [0.07, 0.24]  (4) Differences in mean ranks within each group  (4a) χ2 (2, n = 26) = 35.512 p < 0.01  (4b) χ2 (2, n = 23) = 19.279 p < 0.01 | N/A | The study highlights the enormous possibilities of a toothbrushing application via the smartphone, at least for medium-term oral hygiene improvement in preschool children and even after excluding the app. The long-term effect should also be investigated in a controlled study design to exclude the expected novelty effect. |
| Alklayb et al. (2017),  Saudi Arabia | (1) knowledge score of the mother designed based on a previously validated Arabic tool (based on Farsi et al., 2013)  (2) Improvement in the knowledge of the mothers  before and after the application (Farsi et al., 2013)  (3) Comparison of the improvement of knowledge  between the mothers by region (Paired *t*-test). | **(1) Mean ±SD knowledge scores**  (1a) before using the app: 7.5 **±** 1.6  (1b) after using the app: 14.6 **±** 1.7  (1c) t and p values: -4.232, <0.001  (1d) 95% CI* [6.6, 7.5]  **(2) Mean ±SD improvement in the knowledge scores before and after using the app:**  (2a) Riyadh-Urban: 7.1 **±** 2.6  (2b) Najran-Rural: 6.8 **±** 1.9  (2d) 95% CI* [-0.511, 1.11]  **(3) Change in score (n, mean ± SD)**  (3a) Riyadh-Urban: 616, 7.1 **±** 2.6  (3b) Najran-Rural: 439, 6.8 **±** 1.9  (3c) 95% CI* [-0.517, 1.11]  **(4) Linear regression model** of factors that influence the improvement of maternal knowledge after use of the application  **(Unstandardized coeffiCI*ents [B, SE], Standardized coeffiCI*ents [Beta], t & p values)**  (4a) Constant : [8.746, 0.767], - , 11.406, 0.00  (4b) Age of mother : [-0.047, 0.032],[−0.072], −1.496, 0.135  (4c) No: of children : [0.173, 0.083], [0.099], 2.084, 0.037  (4d) Occupation : [0.042, 0.147], [0.009], 0.287, 0.774  (4e) Income : [2.296E-5, 0.000], [−0.034], −1.089, 0.276  (4f) Region : [−0.450, 0.151], [−0.094], −2.988, 0.003  the mother’s occupation, age, or family income had no significant impact on the change in oral knowledge of the mothers, but the number of children in the family had a significant impact on the knowledge score. | N/A | The research concludes that within the limits of the study, the mobile phone application used in this study improves mothers’ knowledge of their children’s dental health. |
| Alqarni et al. (2018)  Saudi Arabia | (1) Parents’ knowledge about the correct period of baby’s teeth development  (2) Parents’ knowledge of the dental care of children with general health issues | **(1) Parents’ knowledge about the correct period of baby’s teeth development before and after using the app (%)**  (1a) Before using the app(%)  Strongly disagree 20.83  Disagree 28.33  Neither agree nor disagree 25.00  Agree 17.50  Strongly agree 8.33  (1b) After using the app( %)  Strongly disagree 14.16  Disagree 8.33  Neither agree nor disagree 7.50  Agree 26.16  Strongly agree 40.33  **(2) Parents’ knowledge towards the dental care of children with general health issues before and after using the app(%)**  (2a) Before using the app( %)  Strongly disagree 8  Disagree 23  Neither agree nor disagree 31  Agree 28  Strongly agree 10  (2b) After using the app( %)  Strongly disagree 13  Disagree 10  Neither agree nor disagree 14  Agree 34  Strongly agree 29  **(3) Parents’ knowledge about the correct periods of baby’s teeth development.**  χ2 =40.72, P<0.01 HS, Highly significant | N/A | Mobile-based applications are an effective tool for providing child oral health knowledge to parents and significant improvement in knowledge was evident after parents utilised the mobile-based application. |
| Nolen et al (2018)  USA | (1) Prototype Application and the Theory of Planned Behaviour strategies based on Doshi’s intervention strategies (Doshi A et al.,2003)  (2) Prototype Application Usability statements based on a 5-point Likert-scale questionnaire that quantitatively measured perceptions of the app’s interactive design based on Jakob Nielsen’s principles and behavioural strategies (Nielsen J et al.,2016)  (3) Net Promoter Score to measure the likelihood to recommend the app prototype  (4) Audio and video aspects of the app, qualitatively measured using a template approach using codes to identify themes (Faraday J et al.) | **(1) PartiCI*pants mean score on perceptions of TPB Intervention Strategies**  (1a) General Knowledge 5.00  (1b) Increased Knowledge 4.875  (1c) Perceived benefits 5.00  (1d) Perceived Norms 4.875  (1e) Self-efficacy 4.750  (1f) Perceived Norms 2.875  **(2) Application Prototype Usability**  *Usability Statement PartiCI*pant mean score*  (2a) The features of ToothSense, keep parents informed about their child’s oral health and progression towards positive oral health behaviours. 4.645  (2b) Control interactions such as exit, save, go back,  or edit was user friendly. 4.750  (2c) Flexibility and effiCI*ency of the features of  ToothSense 4.125  (2d) App is visually appealing & not overcrowded with  irrelevant information. 3.125  (2e) App provides adequate assistance in recognising, diagnosing, and recovering from errors. 3.500 | The following themes emerged from audio and video recordings:     1. Interface design with sub-theme Aesthetics, size of elements/font, background colour 2. Navigation with subtheme Smiles Club and Mouth Journal functions 3. Feedback with subtheme Photo upload, Smiles Club Interaction 4. Terminology with subtheme Naming of buttons, choice of vocabulary 5. Information with subtheme Oral health information, American Dental Association, dentist approved 6. Health promotion with subtheme Alarm, reminder, timer | Using a mobile platform for oral health promotion provides the opportunity to engage parents, enable communication, and potentially help overcome oral health challenges for the management of oral diseases such as ECC. Beta testing results from this study provided health promotion project design information for the prevention of ECC using the TPB and highlighted the importance of health promotion smartphone app usability. Future research should include modifications to the app based on the prototype test results, pilot testing among parents and caregivers of children 6 years and younger and expansion of the mobile platforms. |
| Lozoya et al. (2019), USA | (1) Participant attitudes, SN, PBC, intentions, or behaviours change after using the smartphone app (paired-sample t-tests).  (2) The mean of each subscale score measured before and after intervention.  (3) Frequency of parents whose scores either changed or did not change from pre- to post-intervention.  (4) thematic analysis, to identify the key themes from the interviews (Ryan GW et al.) | ** PBC=perceived behavioural control, STC=short term consequences, LTC=long term consequences  **(1a) Mean ±SD Pre-intervention scale score (n=26)**  Dietary Attitude 19.07±2.90,  Dietary Norms 56.58±10.11  Dietary PBC 15.27±2.24  Oral Attitudes 15.46±2.37  Oral Norms 54.96±13.11  Oral PBC** 14.92±1.74  Dental Norms 27.85±5.08  Dental PBC** 15.81±1.79  Dental STC** 14.89±2.32  Dental LTC** 8.15±1.16  **(1b) Mean ±SD Post-intervention scale score (n=26)**  Dietary Attitude 18.77±2.34  Dietary Norms 54.96±13.11  Dietary PBC 15.65±1.74  Oral Attitudes 16.08±2.12  Oral Norms 56.58±10.12  Oral PBC 15.00±1.60  Dental Norms 27.77±3.69  Dental PBC** 15.96±2.34  Dental STC** 15.04±2.01  Dental LTC** 8.23±0.86  **(1c) Mean difference & Confidence Interval (CI*)**  Dietary Attitude -0.30, 95% CI* [-0.77, 1.37]  Dietary Norms -1.62, 95% CI* [-3.18, 6.42]  Dietary PBC 0.32, 95% CI* [-0.44, 1.20]  Oral Attitudes 0.62, 95% CI* [-0.29, 1.53]  Oral Norms 1.62, 95% CI* [-3.18, 6.42]  Oral PBC 0.08, 95% CI* [-0.59, 0.75]  Dental Norms -0.08, 95% CI* [-1.75, 1.91]  Dental PBC** 0.15, 95% CI* [-0.70, 1.00]  Dental STC** 0.15, 95% CI* [-0.73, 1.03]  Dental LTC** 0.08, 95% CI* [-0.34, 0.50]  **(1d) Frequency [number(%)] of parents whose scores changed from pre- to post-intervention (n=26)**  (3a) Attitude  *Dietary*: No change 6(23.1), Change 20(76.9)  *Oral (no change)*: No change 8(30.8), Change 18(69.2)  (3b) Perceived Behavioural Control  *Dietary* : No change 9(34.6), Change 17(65.4)  *Oral*: No change 9(34.6), Change 17(65.4)  *Dental Attendance* : No change 10(38.5), Change 16(61.5)  (3c) Intention Change  *Sugary Snacks* :No change 19(73.1), Change 7(26.9)  *Brushing (no change)* :No change 20(76.9),  Change 6(23.1)  *Dental attendance (no change)* :No change 21(80.8),  Change 5(19.2)  (3d) Behaviour Change  *Snacks (no change)* :No change 19(73.1), Change 7(26.9)  *Sugary Drinks (no change)* :No change 16(61.5),  Change 10(38.5)  *Brushing frequency (no change)*:No change 17(65.4),  Change 9(34.6) | The second phase of the study resulted in five emergent themes organised across the Perceived Behavioural Control (PBC) constructs for attitude, Subjective Norms, intentions and PBC ( & Smartphone oral hygiene applications). | Although the results from this study did not support the use of a smartphone app to improve attitudes, SN, intentions, and PBC of the parents of pre-schoolers, it can be concluded that PBC is a significant predictor of dietary, oral hygiene, and dental attendance intentions and should be considered when developing oral health promotion. Social norms were shown to be significant predictors of dietary behaviours and oral hygiene intentions and the use of TPB can support oral health goals in developing oral health promotions aimed at parents of pre-schoolers. Although the qualitative data suggest that a smartphone app supports parents’ efforts to make oral health recommendations part of their pre-schoolers’ daily routine, more quantitative data needs to be collected to establish the use of TPB in developing oral health interventions. |
